# Short-term hyperoxia-induced functional and morphological changes in rat hippocampus

**DOI:** 10.3389/fncel.2024.1376577

**Published:** 2024-04-15

**Authors:** Alexandra Julia Hencz, Andor Magony, Chloe Thomas, Krisztina Kovacs, Gabor Szilagyi, Jozsef Pal, Attila Sik

**Affiliations:** ^1^Institute of Physiology, Medical School, University of Pécs, Pécs, Hungary; ^2^Institute of Clinical Sciences, College of Medical and Dental Sciences, University of Birmingham, Birmingham, United Kingdom; ^3^Institute of Biochemistry and Medical Chemistry, Medical School, University of Pécs, Pécs, Hungary

**Keywords:** hippocampus, hyperoxia, dark neuron, electrophysiology, network oscillation

## Abstract

Excess oxygen (O_2_) levels may have a stimulating effect, but in the long term, and at high concentrations of O_2_, it is harmful to the nervous system. The hippocampus is very sensitive to pathophysiological changes and altered O_2_ concentrations can interfere with hippocampus-dependent learning and memory functions. In this study, we investigated the hyperoxia-induced changes in the rat hippocampus to evaluate the short-term effect of mild and severe hyperoxia. Wistar male rats were randomly divided into control (21% O_2_), mild hyperoxia (30% O_2_), and severe hyperoxia groups (100% O_2_). The O_2_ exposure lasted for 60 min. Multi-channel silicon probes were used to study network oscillations and firing properties of hippocampal putative inhibitory and excitatory neurons. Neural damage was assessed using the Gallyas silver impregnation method. Mild hyperoxia (30% O_2_) led to the formation of moderate numbers of silver-impregnated “dark” neurons in the hippocampus. On the other hand, exposure to 100% O_2_ was associated with a significant increase in the number of “dark” neurons located mostly in the hilus. The peak frequency of the delta oscillation decreased significantly in both mild and severe hyperoxia in urethane anesthetized rats. Compared to normoxia, the firing activity of pyramidal neurons under hyperoxia increased while it was more heterogeneous in putative interneurons in the cornu ammonis area 1 (CA1) and area 3 (CA3). These results indicate that short-term hyperoxia can change the firing properties of hippocampal neurons and network oscillations and damage neurons. Therefore, the use of elevated O_2_ concentration inhalation in hospitals (i.e., COVID treatment and surgery) and in various non-medical scenarios (i.e., airplane emergency O_2_ masks, fire-fighters, and high altitude trekkers) must be used with extreme caution.

## Introduction

Oxygen (O_2_) is the second most abundant element (21%) in Earth’s atmosphere, which plays an essential role in the efficient maintenance of the metabolic processes of animal cells and the normal functioning of all organs (Thannickal, 2009; Raffaella et al., 2016). Oxygen is considered a cornerstone of modern medical care and is often used to reduce the damage caused by hypoxia, especially in the emergency care (Thomas et al., 2022). In clinical practice, normobaric oxygen therapy is used, when high concentrations of oxygen are administered at normal atmospheric pressure. For these therapies, the O_2_ concentration commonly used is between 40% and 100% (Chazalviel et al., 2016; Xu et al., 2016; Gonzales-Portillo et al., 2019). High O_2_ levels are used in the clinic, for example, in anesthesia during surgery, treatment of traumatic brain injuries, in the management of secondary hypoxic brain injury following ischemic stroke, in septic shock, and post-cardiac resuscitation (Calzia et al., 2010; Levy et al., 2016). Oxygen therapy has an important role in newborn care and the treatment of patients with severe respiratory failure (e.g., COPD). Recently, treatment was used for COVID-19 patients in hospitals (Lyons and Callaghan, 2020). In addition, it is increasingly used for preconditioning (even in combination with hypoxia) during sports training, for cardiovascular conditioning, or before extreme environmental stress, such as SCUBA diving, military free-fall or space flight, to reduce the chance of decompression sickness (Webb and Pilmanis, 2011; Balestra et al., 2021; Lafère et al., 2021; Bestavashvili et al., 2022). Furthermore, many researchers point to the potential therapeutic application of oxygen therapy for depression or age-related neurological diseases such as Alzheimer’s disease (Serebrovska et al., 2019; Bloch et al., 2021). One potential application of a hyperoxic environment has also been proposed even in an extraterrestrial application providing a slight increase in the partial pressure of O_2_ on the Moon in underground tunnels to supply O_2_ and compensate for the total pressure (Martin and Benaroya, 2023). Although hyperoxia may seem harmless, measures taken to achieve “adequate tissue oxygenation” due to excess oxygen administration or vasopressor therapy may eventually alter cellular metabolism and functions unintentionally (Maltepe and Saugstad, 2009). Poorly prescribed and poorly administered oxygen is particularly dangerous in critically ill patients (Thomas et al., 2022). High oxygen levels at tissues (hyperoxia) cause oxygen toxicity, which primarily affects the central nervous system, retina, and lungs (Diringer, 2008; Vogel et al., 2015; Lajko et al., 2016). Neurons require a lot of energy because of their electrical activity, so changes in metabolic processes caused by an excess of oxygen play a particularly significant role in the damage of nerve cells (Lassmann and van Horssen, 2016). Sustained high oxygen levels (80% O_2_) in young animals reduce the density of neurons in the hippocampus, subiculum, prefrontal and parietal and retrosplenial cortices (Yiş et al., 2008). A duration of 24–48 h of hyperoxia also reduces both the number of mature and immature neurons, as well as the proliferation of progenitor cells (Endesfelder et al., 2014). It is known that hyperoxia can paradoxically reduce O_2_ delivery due to cerebral vasoconstriction (Watson et al., 2000). Cerebral blood flow and cerebral O_2_ metabolism strongly influence the brain’s electrical activity (Lauritzen et al., 2012). In studies in young rats, 24 h hyperoxia has been shown to reduce the expression of genes involved in acute, subacute, and long-term synaptic processes, including those responsible for the regulation of plasticity (Hoeber et al., 2016). High oxygen levels lasting several days can lead to abnormal neural activity, primarily to a lack of spatial and recognition memory, and even to a smaller size of the hippocampus (Ramani et al., 2013; Lithopoulos et al., 2022). Subregions of the dentate gyrus and the cornu ammonis area 1 (CA1) region of the hippocampus are particularly sensitive to normobaric hyperoxia, hyperoxia increases cell death in these regions (D’Agostino et al., 2007; Yiş et al., 2008; Porzionato et al., 2015). High O_2_ concentrations can negatively affect global protein synthesis and mitochondrial function in the hippocampus, reducing proteins required for hippocampus-dependent learning and memory functions (Ramani et al., 2018). Elevated levels of reactive O_2_ species (ROS) and impaired mitochondrial function may increase the risk of several neurodegenerative disorders such as Alzheimer’s disease (Arendash et al., 2009).

The data in the literature indicate that the oxygen supply exceeding the oxygen demand in the O_2_ metabolism of the brain plays a key role in the damage of the hippocampus through the changes that occur and the impairment of mitochondrial functions. The risk of excess oxygen is determined by many factors, such as the fraction of inspired O_2_ (FiO_2_), partial pressure, and exposure time, and can also be influenced by systemic conditions (Chen et al., 2020). The effect of normobaric hyperoxia on the brain has been studied mainly in the case of longer exposure times and/or severe hyperoxia, but we still have incomplete data on neuronal responses induced by short-term mild hyperoxia.

Therefore, the present study aimed to examine the effect of short-term mild hyperoxia on neuronal viability and network activity in different regions of the hippocampus and to compare it with the effect of short-term severe hyperoxia.

## Materials and methods

### Animals and experimental procedure of hyperoxia exposure

The experiments were performed on male Wistar rats (*n* = 40, Charles River, Hungary) weighing 250–300 g at the time of surgery. The animals were housed in a clean and hygienic environment, on a 12-h light and dark cycle and 23 ± 2°C temperature, and had access to standard laboratory food pellets (CRLT/N Charles River Kft, Budapest, Hungary) and tap water *ad libitum*.

All experimental procedures were performed according to guidelines and protocols approved by the National Ethical Council for Animal Research (Permit number: BA/73/0052-5/2022, Hungary) and the regulations of the European Community Council Directives (Directive 2010/63/EU of the European Parliament and the Council).

Rats were randomly divided into two experimental and control groups: animals were exposed to normoxic (21% O_2_) and hyperoxic (30% and 100% O_2_) conditions at atmospheric pressure. In the induction chamber, the level of O_2_ was continuously monitored with an O_2_ sensor (R17 MED, Viamed Limited, UK). After 1-h O_2_ exposure rats were anesthetized for histological examination by intraperitoneal injection of urethane (1.5–2.0 g/kg, Sigma, St. Louis, MO, USA).

### Silver impregnation method (Gallyas staining)

After 1-h O_2_ exposure, the rats (21% *n* = 10, 30% *n* = 10, and 100% *n* = 10) were anesthetized by intraperitoneal injection of urethane (1.5–2.0 g/kg). Immediately after euthanasia, transcardial perfusion with 4% paraformaldehyde (PFA) in 0.1 M phosphate-buffered saline (PBS) was performed. Brains were excised 12 h after fixation and post-fixed in 4% PFA in PBS. Brain tissues were cut into coronal slices (50 μm) using a vibratome (Vibratome^®^ Series 1000; Technical Products International Inc., St Louis, MO, USA). A special Gallyas silver impregnation method was carried out to detect the compaction of dark neurons. With this staining procedure, the early stages of neuron degeneration can be detected (Gallyas et al., 1990). In brief, brain slices were dehydrated through a series of 1-propanol 50% and 100% (1–2 min) then incubated for 16 h at 56°C in 1-propanol containing 1% sulfuric acid (esterification). Sections were rehydrated in a series of 1-propanol 100% and 50% (1–2 min), followed by washing with double-distilled water for 5 min and treated with 1% acetic acid for 5 min. Slices were stained by silver solution and 1% acetic acid was added to stop the reaction.

### Surgery and electrophysiological recording

For the surgical procedure and electrode implantation, rats (*n* = 10) were anesthetized by intraperitoneal injection of urethane (1.1–1.3 g/kg; Sigma, St. Louis, MO, USA) and fixed in the stereotaxic frame. The O_2_ administration was carried out via an anesthesia mask. A 32-channel silicon probe was implanted for the recording of neuronal activity under sterile conditions. In brief, the skull was exposed and cleaned then a 2 mm hole was drilled over the hippocampus (Hilus-CA1 region: Medial-Lateral 1.2–2.2 mm, Anterior-Posterior −4 mm and CA3 region: Medial-Lateral 3.6–4.6 mm, Anterior-Posterior −4 mm) according to the atlas of Paxinos and Watson (2006). Dura mater was gently removed and the 32-channel multielectrode array (A4×8-5 mm-200-400-703, NeuroNexus Technologies, Inc., USA) was dipped in 2% DiI solution before lowering to the hippocampus. The probes were attached to a micromanipulator (Hilus-CA1 region: Medial-Lateral 1.4–2.0 mm, Anterior-Posterior −4 mm, Dorsal Ventral −3.6 mm, CA3 region: Medial-Lateral 3.8–4.4 mm, Anterior-Posterior −4 mm, Dorsal-Ventral −4 mm) (Paxinos and Watson, 2006).

The O_2_ level in the brain was monitored with a 10 μm diameter, modified Clark-type polarographic O_2_ microelectrode (OX-10, Unisense A/S, Aarhus, Denmark) in the proximity (less than 100 μm) to the silicon probes. The sensor currents were measured with a high-impedance picoammeter (PA 2000, Unisense A/S, Aarhus, Denmark). The microelectrode was calibrated according to the manufacturer’s protocol (see Unisense website). In short, the calibration of the O_2_ microsensor was performed by a conventional two-point calibration in O_2_-free and air-saturated solution.

Local field potential (LFP) was recorded in normoxic (21% O_2_) and hyperoxic (30% and 100% O_2_) conditions. Field potential and unit activity were recorded with an amplifier and referenced to both internal and cranial references. The extracellular recordings were acquired using a 128-channel TDT system (Tucker-Davis Technologies Inc., FL, USA) with a sampling frequency of 12 kHz and a LabChart virtual instrument controlling an analog-to-digital converter card (AD Instruments).

### Microscopy

Olympus BX61 TRF fluorescent microscope (Olympus Corporation, Tokyo, Japan) was used for the collection of images. Gallyas stains were qualitatively analyzed through light microscopy using a halogen bulb. All images were taken at 10× magnification. Counting of dark neurons was performed with the Image-Pro plus 7.0 (Media Cybernetics, Inc., Rockville, MD, USA, 2009) software. Areas of hippocampal regions (CA1, CA3, and dentate hilus) were measured with Image-Pro plus 7.0 and the density of stained cells were calculated per 10,000 μm^2^. Because of the extreme low density value in normoxia in all regions, these values are taken as 0.

### LFP data processing

Local field potential recordings were subjected to spectral analysis using built-in functions in Matlab (The MathWorks, Inc., Natick, MA, USA). To isolate single unit activity and detect firing rate and interspike interval (ISI) values, the online algorithm of the recording software with a bandpass filter of 500–5,000 Hz was used. We used a bandpass filter to decompose the data into delta (1–4 Hz), theta (4–8 Hz), alpha (8–12 Hz), beta (12–30 Hz), and gamma (30–100 Hz) bands. The anatomical position of each recording channel was determined based on the spatial location of electrodes, the distance between the recording channels, and the amplitude and orientation of the theta waves. Only unit activities with a stable spike waveform during the recording period, from 21% O_2_ to 30% O_2_ and finally to 100% O_2_ exposure, were included in the analysis. The physical location of recording channels, firing frequency and interspike interval values were used to separate pyramidal cells from inhibitory cells. Pyramidal cells are generally characterized by firing at a low frequency (<5 Hz). Compared to excitatory pyramidal cells, inhibitory interneurons discharge at a high rate (>5 Hz) (Csicsvari et al., 1999; Klausberger et al., 2003). For ISI values, the cut-off point for distinguishing between putative pyramidal cells and inhibitory neurons was marked at 200 ms in the pyramidal cell layers. The standard deviation (SD) values were computed from the series of ISI values for each recording channel.

### Statistical analysis

Statistical tests and graphs were completed with SPSS 28 (SPSS Inc., Chicago, IL, USA) and Microsoft Excel 365 (Microsoft Inc., Redmond, WA, USA). Data distributions were tested with a histogram and the Shapiro–Wilk test. If the data were normally distributed, one-way ANOVA with Tukey’s *post-hoc* test was applied for multiple group comparison. If data were non-normally distributed, a Kruskal–Wallis test and Dunn’s multiple comparisons were performed for histological data, and related samples Friedman’s two-way analysis for unit activity data. Results were represented as mean ± SEM and *p* < 0.05 was considered significant.

## Results

### Hyperoxia increases the number of dark neurons in the hippocampus

**FIGURE 1:**
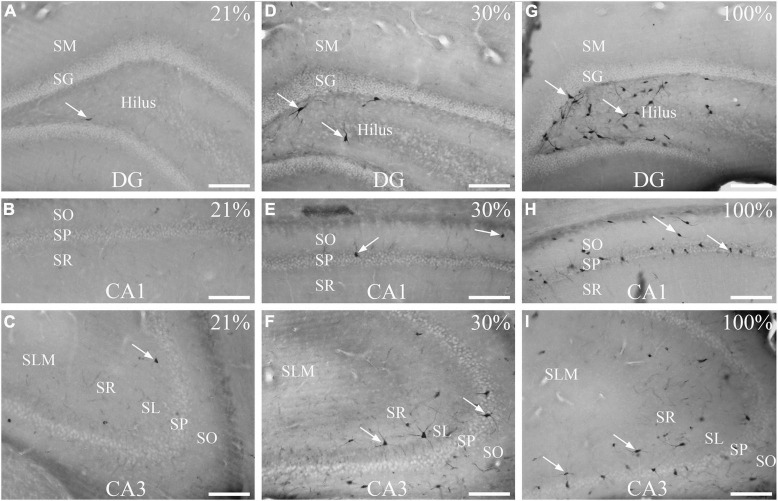
Effects of normoxia (21% O_2_), 30%, and 100% hyperoxia on the number of dark neurons after 1 h exposure. **(A)** Only one dark neuron (arrow) is visible in the hilus. **(B)** Silver impregnation staining shows no dark neurons in the CA1 region. **(C)** There are few dark neurons in the CA3 region (arrow). **(D)** Silver-impregnated dark neurons can be seen in the subgranular zone of the dentate gyrus and the deep hilus (arrows) after 30% O_2_ exposure. **(E)** Dark neurons in the str. oriens and str. pyramidale of the CA1 region (arrows) in 30% O_2_ treated animals. **(F)** In the CA3 area, many dark neurons with stained neurites are visible (arrows) after mild (30%) hyperoxia. Silver impregnation staining shows numerous dark neurons in the subgranular zone of the dentate gyrus, in the deep hilus (**G**, examples pointed with arrows), and in the CA1 **(H)** and CA3 regions **(I)**. SO, stratum oriens; SP, stratum pyramidale; SR, stratum radiatum; SL, stratum lucidum; SLM, stratum lacunosum-moleculare; SM, stratum moleculare; SG, stratum granulare; DG, dentate gyrus. Scale bar: 100 μM.

**FIGURE 2:**
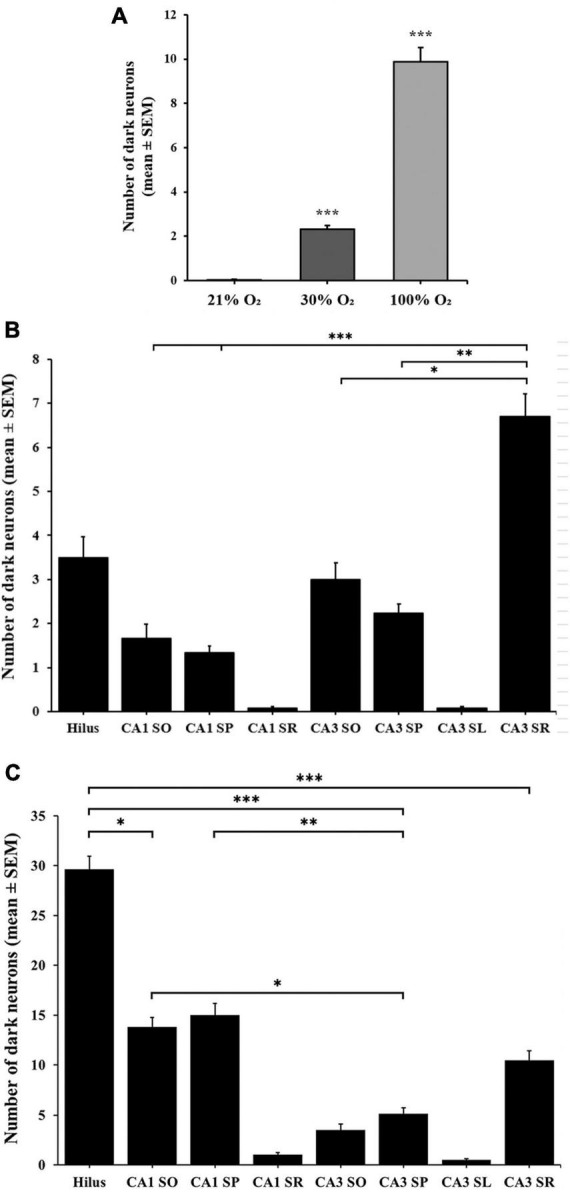
Number of dark neurons in the hippocampus after 1-h exposure to 30% and 100% O_2_. **(A)** The 30% and 100% experimental groups show a statistically significant difference in the number of silver-impregnated dark neurons compared to the control (21%) group and each other. **(B)** The total number of dark neurons was higher in the layers of the CA3 region in the 30% O_2_ group than in the hilus and CA1 regions. **(C)** The number of dark neurons significantly increased in the 100% O_2_ group. In the hilus, more stained neurons were observed than in the layers of the CA1 and CA3 regions. *n* = 30 slices in each group. Data are presented as mean ± SEM. *p < 0.05, **p < 0.005, ***p < 0.001, Kruskal–Wallis test followed by Dunn’s multiple comparisons test.

To induce hyperoxia in rats, animals were exposed to 30% and 100% O_2_ for 1 h (*n* = 10 animal/group). Silver impregnation staining was performed to quantify the number of stained, dark neurons of the hilus, CA1 and CA3 hippocampal regions. We found very few (1–2) dark neurons in some control animals due to effects not yet determined (Figures 1A–C). The number of stained neurons slightly increased in the hippocampus (2.32 ± 0.17) after 1-h of mild (30% O_2_) hyperoxic treatment, compared to the control (*p* < 0.005, Figure 2). Dark neurons were observed in the deep hilus (3.50 ± 0.47, hilus: 0.9/10,000 μm^2^) and the subgranular zone of the dentate gyrus (Figure 1D). In the CA1 region (CA1: 0.1/10,000 μm^2^) dark neurons were detected in the stratum oriens (1.66 ± 0.32), stratum pyramidale (1.33 ± 0.15), and stratum radiatum (0.06 ± 0.04) layers (Figure 1E). In the CA3 region (0.4/10,000 μm^2^) dark neurons were found in the stratum oriens (3.00 ± 0.37), stratum pyramidale (2.30 ± 0.21), stratum lucidum (0.06 ± 0.04), and stratum radiatum (6.70 ± 0.59) layers (Figure 1F). The number of dark neurons significantly increased in the stratum radiatum of the CA3 region compared to cell layers of the CA1 region (*p* < 0.001, Figure 2B). Similarly, dark neurons were detected after the 100% O_2_ exposition, but the number of dark neurons markedly increased (9.88 ± 0.66) compared to the control and 30% O_2_ groups (*p* < 0.005, Figure 2A). Stained neurons appeared in the deep hilus (29.63 ± 1.33, hilus: 2.1/10,000 μm^2^) and the subgranular zone of the dentate gyrus (Figure 1G). In the hilus were observed significantly more stained neurons than in the layers of the CA1 (0.3/10,000 μm^2^) and CA3 regions (*p* < 0.05, 0.6/10,000 μm^2^, Figure 2C). In the CA1 region, silver-stained dark neurons were located in the stratum oriens (13.80 ± 0.97), stratum pyramidale (15.00 ± 1.17), and stratum radiatum (1.03 ± 0.21) layers (Figure 1H). In the CA3 region, most dark neurons were observed in the stratum radiatum (10.43 ± 0.96), while fewer damaged neurons were in the stratum oriens (3.46 ± 0.61), stratum pyramidale (5.13 ± 0.60), and stratum lucidum (0.50 ± 0.13) (Figures 1I, 2C). No dark neurons were found in the stratum lacunosum-moleculare layer in any of the O_2_ exposures. The sample processing was the same in the different groups, so it can be assumed that the number of dark neurons reflects the effect of the elevated O_2_ concentration in the mild and severe hyperoxia groups. Moreover, the data show that among the hippocampus regions, the neurons of the hilus are more sensitive in severe hyperoxic conditions.

### Hyperoxia decreases the low-frequency hippocampal activity in urethane-anesthetized rats

The effect of hyperoxia on brain activity was investigated by measuring field potentials derived from the hippocampus layers under urethane anesthesia (*n* = 9 rats). The brain state exhibited stable activity during the initial control run for 60 min followed by 15 min of hyperoxic exposure. A modified Clark-type polarographic O_2_ microelectrode was used to directly measure the O_2_ level of the hippocampus. The O_2_ sensor was placed near the multichannel array. During the baseline recording, the partial pressure was measured at 20.1 ± 4.36 mmHg. At 30% O_2_ inhalation, the partial pressure in the hippocampus tissue increased to 42.4 ± 12.94 mmHg. During the application of 100% O_2_, the partial pressure increased further to 79.04 ± 16.20 mmHg.

**FIGURE 3:**
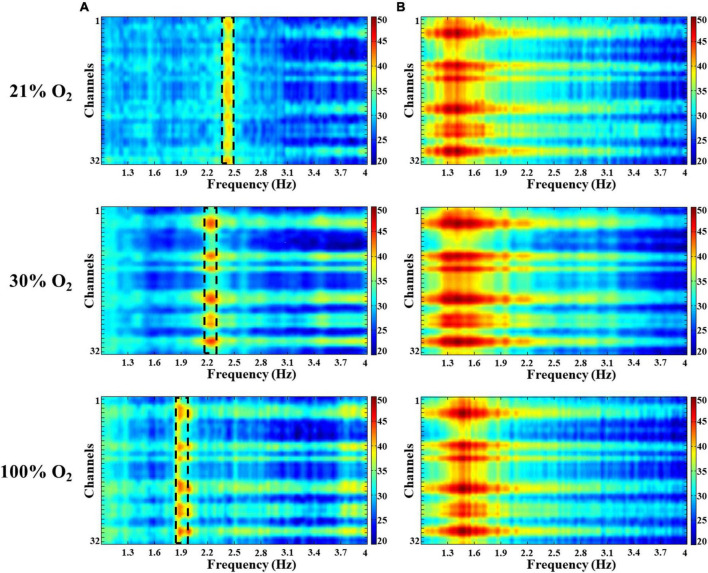
Spectral characteristics of the delta and theta activity in the hippocampus in hyperoxia. Colors represent spectral power ranging from blue (low) to red (high) on a common scale (20–50 dB/Hz). **(A)** A slow oscillation appeared around 2 Hz in the delta band and decreased with increasing O_2_. **(B)** No significant changes were observed in the theta band during hyperoxia.

**FIGURE 4:**
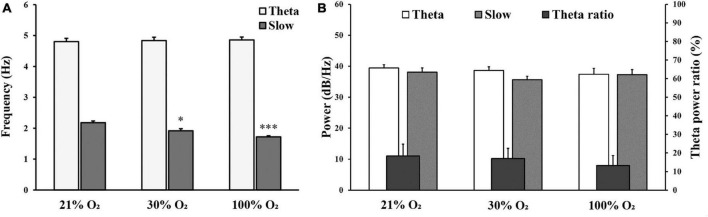
**(A)** Comparison of frequency (Hz) for theta oscillation and slow component at 21%, 30%, and 100% O_2_ concentrations. **(B)** Comparison of spectral power (dB/Hz) for theta oscillation, slow component, and the ratio of theta/delta power in normoxia and hyperoxia. *n* = 9 rats. Data are presented as mean ± SEM. *p < 0.05, *** *p* < 0.001, one-way ANOVA followed by Tukey’s *post-hoc* test.

We observed no significant changes in alpha (8–12 Hz), beta (12–30 Hz), and gamma (30–100 Hz) oscillations under hyperoxic conditions (not shown). The baseline recordings showed in the delta band (0.5–4 Hz) a distinct, slow-frequency activity around 2 Hz, which has the activity altered under hyperoxia (Figure 3A). By increasing the O_2_ concentration to 30%, the peak frequency significantly decreased (1.92 ± 0.07 Hz) in comparison to baseline recordings (2.18 ± 0.05 Hz, *p* < 0.05). In addition, this slow oscillation reduced (1.72 ± 0.05 Hz) after 100% O_2_ exposure and the frequency peak was significantly different compared to the baseline (*p* < 0.001, Figure 4A). We compared the peak frequency of slow- and theta activity (Figures 3A, B, 4A) and the characteristics of theta oscillation did not change significantly (4.80 ± 0.11 Hz vs. 4.84 ± 0.11 Hz and 4.86 ± 0.10 Hz, normoxia vs. 30% O_2_ and 100% O_2_, *p* = 0.936) as a result of O_2_ administration. Spectral power was also examined and there were no significant changes in slow component spectral power values (38.08 ± 1.37 dB/Hz vs. 35.63 ± 1.15 dB/Hz and 37.31 ± 1.63 dB/Hz, normoxia vs. 30% O_2_ and 100% O_2_, *p* = 0.456) or theta oscillation spectral power values (39.44 ± 1.05 dB/Hz vs. 38.64 ± 1.20 dB/Hz and 37.42 ± 1.91 dB/Hz, normoxia vs. 30% O_2_ and 100% O_2_, *p* = 0.612, Figure 4B). We also checked whether the theta ratio changed due to hyperoxia. We found no significant difference in the ratio (18.40% ± 6.39% in normoxia, 16.97% ± 5.50% in 30% O_2_, and 13.27% ± 5.40% in 100% O_2_; Figure 4B).

In summary, we found a selective and significant shift in the frequency of a slow field potential activity, suggesting that O_2_ may be responsible for the shift toward lower frequencies during hyperoxia.

### Hyperoxia increases the firing activity of pyramidal neurons

To determine how hyperoxia changes the activity of individual hippocampal neurons, interneuron and pyramidal cell unit activity was separated based on the anatomical location of recording channels, firing frequency and inter-spike interval values. The unit activities were recorded first in normoxic conditions and then at 30% and 100% O_2_ concentrations. Only those neuron activities were analyzed of which neurons we were able to keep during the whole protocol (21%, 30%, and 100% O_2_). The hippocampus O_2_ level was also measured near the recording electrodes during electrophysiology recording to monitor the state of oxygenation of the brain tissue not just only the inhaled O_2_ concentration.

**FIGURE 5:**
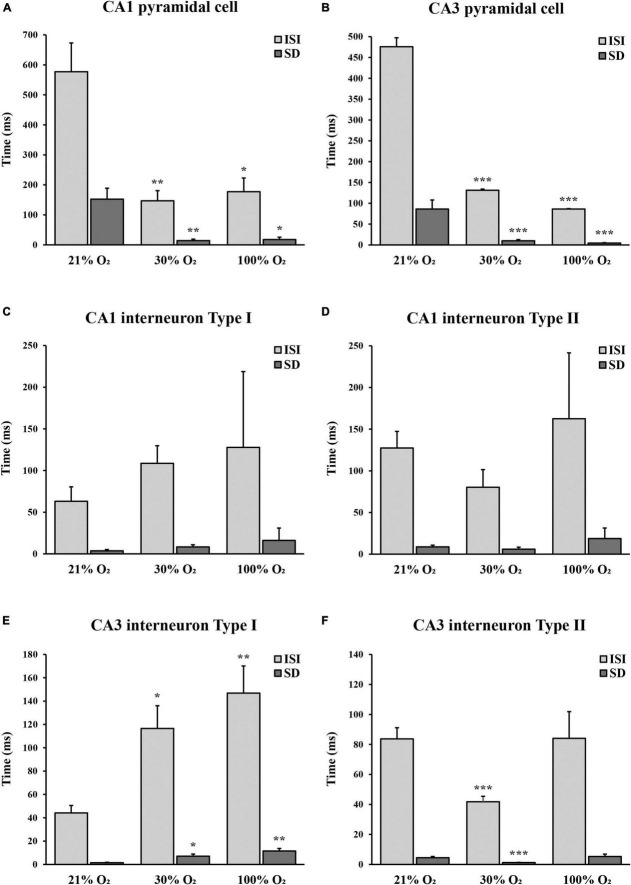
The unit activity of hippocampal CA1 and CA3 pyramidal cells and interneurons in normoxic and hyperoxic (30% and 100% O_2_) conditions. **(A)** Inter-spike interval (ISI) and standard deviation (SD) values significantly decreased in CA1 pyramidal cells both at 30% and 100% O_2_ exposure (*n* = 9). **(B)** Administration of 30% and 100% O_2_ resulted in a significant decrease in the ISI and SD values in CA3 pyramidal cells (*n* = 20). **(C)** Inter-spike interval and SD values did not change significantly in the CA1 type I interneurons in hyperoxic conditions (*n* = 12). **(D)** No difference in the ISI and SD values was observed in the CA1 type II interneurons in hyperoxia (*n* = 7). **(E)** Inter-spike interval and SD values significantly increased in CA3 type I interneurons both at 30% and 100% O_2_ exposure (*n* = 12). **(F)** Administration of 30% O_2_ led to a significant decrease of ISI in CA3 type II interneurons, while ISI and SD values were not different after 100% O_2_ exposure (*n* = 29). Data are represented as mean ± SD. *p < 0.05, **p < 0.005, ***p < 0.001, related-samples Friedman’s two-way analysis.

In the CA1 region, nine pyramidal cells were analyzed with a mean ISI value of 577.27 ms (SEM = 95.71) and mean SD of 152.43 ms (SEM = 36.33) at 21% O_2_ concentration, compared to the mean ISI of 147.05 ms (SEM = 33.63) and mean SD of 14.10 ms (SEM = 4.94) at 30% O_2_ concentration and to the mean ISI of 177.40 ms (SEM = 45.51) and mean SD of 17.92 ms (SEM = 7.68) at 100% O_2_ concentration. The firing activity of the pyramidal cells significantly increased in CA1 at 30% and 100% O_2_ exposure (30% O_2_, *p* = 0.003 and 100% O_2_, *p* = 0.007). Furthermore, we found a significant decrease in SD value at 30% O_2_ (*p* = 0.001) and 100% O_2_ concentration (*p* = 0.014, Figure 5A). This means that hyperoxia increases firing frequency and regularity in CA1 pyramidal cells.

Twenty pyramidal cells were found in the CA3 region with a mean ISI value of 475.82 ms (SEM = 55.88) and mean SD of 86.35 ms (SEM = 21.60) in normoxia, compared to the mean ISI of 131.23 ms (SEM = 24.84) and mean SD of 9.98 ms (SEM = 2.96) at 30% O_2_ concentration and to the mean ISI of 86.37 ms (SEM = 12.84) and mean SD of 4.60 ms (SEM = 0.88) while 100% O_2_ was supplied. We detected a significant decrease in the ISI value when the O_2_ level was increased from 21% to 30% (*p* < 0.001) and from 30% to 100% (*p* < 0.001). In addition, SD values significantly were lower during 30% O_2_ (*p* < 0.001) and 100% O_2_ (*p* < 0.001) exposures (Figure 5B). These results show that hyperoxia increases firing frequency and regularity in CA3 pyramidal cells.

### Hyperoxia changes the firing activity of inhibitory neurons

Twelve putative interneurons were recorded in the CA1 region during 21%, 30%, and 100% O_2_ application, which were divided into two groups based on the tendency of firing frequency change in hyperoxia (Table 1). The first putative interneuron group (*n* = 5) had a mean ISI value of 63.04 ms (SEM = 3.62) and mean SD of 17.37 ms (SEM = 1.58) during normoxia, compared to the mean ISI of 108.70 ms (SEM = 8.37) and mean SD of 21.08 ms (SEM = 2.53) at 30% O_2_ concentration and to the mean ISI of 127.76 ms (SEM = 16.11) and mean SD of 91.00 ms (SEM = 14.85) at 100% O_2_ concentration. No statistical differences were found in the ISI value (*p* = 0.091) and SD value (*p* = 0.247) of putative CA1 interneurons in response to excess O_2_ (Figure 5C). Based on the results, the frequency of action potential of putative CA1 type I interneurons decreased but not significantly in hyperoxia. Likewise, the second group (type II, *n* = 7) mean ISI (127.40 ± 8.72 ms) value did not change significantly (*p* = 0.066) neither in mild hyperoxia (80.25 ± 5.94 ms) nor severe hyperoxia (162.53 ± 18.70 ms). Moreover, there is no significant difference in SD between normoxic (19.94 ± 2.07 ms) and hyperoxic conditions (21.09 ± 2.47 and 79.06 ± 12.68 ms, respectively, *p* = 0.180, Figure 5D).

**TABLE 1:** Summary of neuronal firing rates in normoxic and hyperoxic conditions.

Neuron type	21% O_2_ ISI (mean + SD) ms	30% O_2_ ISI (mean + SD) ms	100% O_2_ ISI (mean + SD) ms	Frequency change in hyperoxia	Firing regularity change in hyperoxia
CA1 pyramidal (*n* = 9)	577.27* ± 152.43*	147.05* ± 14.10*	177.40 ± 17.92	Increase	Regular
CA1 interneuron type I (*n* = 5)	63.04 ± 17.37	108.70 ± 21.08	127.76 ± 91.00	No change	No change
CA1 interneuron type II (*n* = 7)	127.40 ± 19.94	80.25 ± 21.09	162.53 ± 79.06	No change	No change
CA3 pyramidal (*n* = 20)	475.82* ± 86.35*	131.23* ± 9.98*	86.37* ± 4.60*	Increase	Regular
CA3 interneuron type I (*n* = 12)	44.19* ± 1.49	116.57* ± 7.12*	146.83* ± 11.57*	Decrease	Irregular
CA3 interneuron type II (*n* = 29)	83.70* ± 4.49*	41.80* ± 1.23*	84.07 ± 5.25	Increase	Regular

Asterisks represent significant differences between the conditions. A decrease in SD means more regular activity of neurons.

In the CA3 region, we found 41 putative interneurons with stable recording, which were divided into two groups. In the first putative interneuron group (*n* = 12) the mean ISI was 44.19 ms (SEM = 6.35) and the mean SD was 1.49 ms (SEM = 0.38) under normoxic condition, compared to the mean ISI of 116.57 ms (SEM = 19.46) and mean SD of 7.12 ms (SEM = 1.77) at 30% O_2_ concentration and to the mean ISI of 146.83 ms (SEM = 23.30) and mean SD of 11.57 ms (SEM = 2.15) at 100% O_2_ concentration. The mild hyperoxia and severe hyperoxia significantly increased the ISI (*p* = 0.024 and *p* = 0.002, Figure 5E). Similarly, the value of SD significantly increased during mild (*p* = 0.024) and severe (*p* = 0.002) hyperoxic conditions. Results show that hyperoxia decreases firing frequency and regularity in putative CA3 type I interneurons (Table 1).

In contrast to the first interneuron group, in the second putative interneuron group (*n* = 29) mean ISI value (83.70 ± 7.44 ms) and mean SD (4.49 ± 0.81 ms) significantly reduced to the ISI of 41.80 ms (SEM = 3.64, *p* < 0.000) and SD to 1.23 ms (SEM = 0.17, *p* = 0.005) when the O_2_ concentration was increased from 21% to 30%. However, there is no significant difference in the ISI (84.07 ± 17.79 ms, *p* = 0.627) and SD (5.25 ± 1.62 ms, *p* = 0.642) after 100% O_2_ exposure (Figure 5F). Thus, the frequency of action potential of putative CA3 type II interneurons significantly increased only at 30% O_2_ level.

To summarize unit activity results, the firing activity of pyramidal cells increased, but the activity of putative interneurons showed a more diverse response both in the CA1 and CA3 regions during hyperoxic conditions. A significant difference was detected in the CA3 region, where one group of putative interneurons (type I) decreased the firing activity during O_2_ administration, while in the other group (type II) we observed an increase in the firing activity only at 30% O_2_ concentration. By contrast, the change in the firing activity of putative interneurons was not significant in the CA1 region during the application of O_2_. The change in SD of ISI represents the regularity of the firing of neurons. We found that when O_2_ concentration is increased, CA1 and CA3 pyramidal cells firing patterns become more regular. This was also observed for the putative CA3 type II interneurons at 30% O_2_ concentration. Nevertheless, the activity of putative CA3 type I interneuron and CA1 interneuron groups showed increased irregularity during rising O_2_ levels.

## Discussion

In the present study, we investigated the effect of short-term normobaric hyperoxia on the morphology and function of the hippocampal neurons in rats. We showed that hyperoxic expositions (both 30% and 100% O_2_) increased the number of dark neurons in different hippocampal regions. By examining neuronal activity using a multielectrode array, we found a low-frequency activity in the delta frequency range decreased with the increase of O_2_ concentration. Furthermore, we observed that the firing frequency of pyramidal cells increased, but putative interneurons showed functional heterogeneity in both CA1 and CA3 regions in hyperoxia.

To our knowledge, the occurrence of dark neurons is reported for the first time in the brain after hyperoxic exposure in this present study. Silver-impregnated neurons have been previously detected in hypoxia, ischemia, epilepsy, hypoglycemia, hyperglycemia, and traumatic brain injury (Auer et al., 1985; Pál et al., 2006; Kövesdi et al., 2007; Gallyas et al., 2008; Ahmadpour and Haghir, 2011; Mahakizadeh et al., 2020; Hencz et al., 2023). Mahakizadeh et al. (2020) observed dark neuron production in both CA1 and CA3 regions in chronic hypoxia (Mahakizadeh et al., 2020). Our previous research has described that short-term mild hypoxia causes dark neuron formation in the CA1 and CA3 regions and the hilus (Hencz et al., 2023). Dark neurons have several morphological characteristics, such as massive shrinkage, high electron density, hyperbasophilia and induced hyperargyrophilia (Gallyas et al., 2004). The presumed mechanism of the ultrastructural compaction behind the change in the intracellular structures is the release of the free energy stored in a metastable state of individual neurofilaments at any point. The released energy serves as activation energy at the neighboring points and spreads in the cytoskeletal network (Gallyas et al., 2004). Based on previous studies, excitatory neurotransmitters like glutamate and free radicals may have a role in the initiation of the contractile process during dark neuron formation (Vohra et al., 2002; Kherani and Auer, 2008). The used Gallyas silver method is practically free from the staining of normal structures of neurons, therefore the rapid occurrence of hyperargyrophilic phenomenon shortly after the external initialization indicates the degradation of the affected cells (Kawai et al., 1992; Csordás et al., 2003; Uchihara, 2007). Dark neurons are morphologically similar to apoptotic cells, but dark neurons may spontaneously recover, which probably depends on the extent and time of the damage (Csordás et al., 2003; Gallyas et al., 2006; Kövesdi et al., 2007; Toth et al., 2016).

Several previous studies have shown that supraphysiological O_2_ concentrations cause neuronal damage upon reperfusion and in the brain of developing animals (Felderhoff-Mueser et al., 2004; Shimabuku et al., 2005; Gerstner et al., 2008; Koch et al., 2008; Brücken et al., 2010). For instance, hyperoxia-induced neurodegeneration has been demonstrated in the developing rat brain, where the density of neurons decreased in the dentate gyrus and CA1 regions of the hippocampus, as well as in the prefrontal cortex, parietal cortex, subiculum, and retrosplenial cortex after 5-day 80% O_2_ treatment (Yiş et al., 2008). Similarly, apoptotic cell death and superoxide anion level increase were observed in CA1 neurons of rat hippocampal slices when the O_2_ concentration was 95%, but there was no significant difference at O_2_ levels of 60% and below (D’Agostino et al., 2007). In the present study, we did not observe apoptosis after 1-h hyperoxic treatment (TUNEL assay, data not shown), but our results suggest that mild hyperoxia leads to neuronal damage not only in the CA1 and hilus but also in the CA3 region. We assume that increased ROS and reactive nitrogen species levels due to excessive O_2_ supply play a significant role in the damage. Reactive O_2_ species are also produced throughout normal metabolic reactions, including anaerobic respiration at the electron transport chain within the mitochondria, as well as reactions of cyclooxygenases, lipoxygenases, peroxidases, and cytochrome P450 enzymes (Kirsch and de Groot, 2000). Nevertheless, the high O_2_ concentration disrupts the pro-oxidant-antioxidant balance, leading to general oxidative damage of DNA, lipids and proteins (Ottolenghi et al., 2020; Juan et al., 2021; Leveque et al., 2023).

A recent study investigated the brain’s susceptibility to oxidative stress induced by short-term hyperoxia treatment in adult Wistar rats. Hyperoxia at a FiO_2_ of 60% was shown to cause significant oxidative damage to hippocampal lipids and proteins after 2 h of oxygenation, but not at a FiO_2_ of 40% (Machado et al., 2022). Furthermore, in a study of human subjects, researchers found that the levels of oxidation markers were increased in the first 8 h after exposure to 1 h normobar hyperoxia (30% and 100%), and the subsequent inflammatory response was significantly higher at FiO_2_ of 100% (Leveque et al., 2023). In our study, we hypothesized that the difference between the hippocampal regions observed at 30% and 100% O_2_ concentrations may arise from cell type-specific differences in terms of sensitivity to ROS.

Both normobaric and hyperbaric hyperoxia are frequently used in various clinical scenarios and O_2_ therapy, therefore investigating the effect of elevated O_2_ on network activity is important. Several early human studies have reported the effect of hyperoxia on neural activity, but these results are contradictory, mainly due to the different conditions during recordings. In previous studies, it has been shown that elevated O_2_ did not alter resting-state EEG or evoked potentials (Smith and Strawbridge, 1974; Kaskinoro et al., 2010). In contrast, Sheng et al. (2017) found that hyperoxia decreased the alpha and beta band power of spontaneous neural activity and that certain peaks of visual stimulation event-related potentials were delayed during 98% hyperoxic treatment (Sheng et al., 2017). Another research study reported that during short-term exposure to 100% O_2_, alpha, beta and gamma frequencies of brain activities decreased in eyes-open resting states, while during eyes-closed conditions hyperoxia decreased the oscillation in the beta range with a concomitant increase in both delta and theta power (Kizuk et al., 2019). Similarly, supplementation with 35% O_2_ resulted in a considerable bilateral increase in delta power and a significant bilateral decrease in beta and gamma power (Seo et al., 2007). In our study, we did not observe significant changes in the theta, beta and gamma oscillations of urethane anesthetized rats. However, it is important to note that blood flow and O_2_ regulation may differ between awake and anesthetized animals. In the brains of awake mice exposed to hyperoxia, it was shown that interstitial PaO_2_ is higher under isoflurane anesthesia than in the awake state (Lyons et al., 2016). This is probably related to the vasodilatory effects of isoflurane, which affects cerebrovascular activity in a dose-dependent manner (Farber et al., 1997; Lyons et al., 2016).

In contrast, other anesthetics, such as urethane, have minimal effects on the cardiovascular system, but an increase in dose leads to reduced blood flow (Iwamoto et al., 1987; Sakaeda et al., 1998). The state of the brain can be directly or indirectly affected by changes in breathing rate, O_2_ concentration and CO_2_ level (Pappenheimer, 1977; McQuillen and Ferriero, 2004; Kwak et al., 2006; Zappe et al., 2008; Ito et al., 2013; Pagliardini et al., 2013; Hauer et al., 2018). In this experiment, we found that delta wave frequency (∼2.4 Hz) decreased with increasing O_2_ concentration (30% then 100% O_2_) under urethane anesthesia. This is consistent with previous results demonstrating that exposure to hyperoxia shifts the brain toward slow-wave states during urethane anesthesia and the natural sleep (Hauer et al., 2018). However, we observed no changes in the power values of the slow oscillation band during the hyperoxic exposures. The delta oscillation originates from the thalamic neurons and the deep cortical layers (Dossi et al., 1992; Steriade et al., 1993). In the case of delta wave activity, several studies have demonstrated a direct relationship between blood flow and delta wave band power. The delta wave activity increases during the blockage of the blood flow (ischemic stroke), which may represent the sustained membrane hyperpolarization and inhibition of cortical neurons (John and Prichep, 2006; Foreman and Claassen, 2012; Fanciullacci et al., 2017; Ferreira et al., 2021). Reduced blood flow can also develop during hyperoxia as the systemic effects of hyperoxia include a decrease in blood flow in the brain, coronary and vascular systems (Thomas et al., 2022). Pyramidal neurons found in layers III, V, and VI are extremely sensitive to reduced blood flow, thus leading to many abnormal EEG changes (Jordan, 2004). The neocortical neuronal discharges influence hippocampal network activity via the entorhinal input (Sirota et al., 2003), thereby presumably producing diverse patterns in the hippocampal delta waves of sleep in altered O_2_ conditions. According to previous results on hypoxia, an O_2_ concentration slightly lower than the physiological condition causes an increase in the delta wave activity (Pappenheimer, 1977; Hamrahi et al., 2001; Hoshikawa et al., 2014; Hencz et al., 2023). Our results suggest that the reduction of frequency in slow waves under hyperoxia is related to the secondary effects of O_2_ that may affect cerebral metabolism.

It is assumed that O_2_ has a direct effect on the functioning of mitochondria and can cause mitochondrial dysfunction in neurons and glial cells, on the other hand, the increase in intracellular Ca^2+^ level induced by ROS affects the signaling pathways of astrocytes. Our result showed that spike activity increased both in the CA1 and CA3 pyramidal neurons at 30% and 100% hyperoxic exposure, respectively. A previous study showed that hyperbaric hyperoxia impairs neuronal excitability and synapses, but this may be primarily due to the sensitivity of excitability cells to barometric pressure (Torbati et al., 1976; Colton and Colton, 1982; Garcia et al., 2010a,b). For instance, in the CA1 region, a single transient acute hyperbaric hyperoxia stimulus increases neuronal excitability and stimulates neural plasticity in a wide range of tissue O_2_ tensions (Garcia et al., 2010a). In our case, the external pressure had no stimulating effect during the experiment suggesting that the sensitivity of pyramidal cells and interneurons can be attributed to the secondary effects of O_2_. The molecular O_2_ may interact with lipid-lipid or lipid-protein in the plasma membrane, resulting in decreased fluidity of the plasma membrane (Bennett et al., 1967; Block et al., 1986; D’Agostino et al., 2009). As a result, ion channels and membrane proteins in general react sensitively to changes in the membrane composition (Tillman and Cascio, 2003). Therefore, the alterations of ion channel characteristics or channel expression can affect the neuronal excitability (Noda et al., 1983; Gu and Haddad, 2003; Mulkey et al., 2003; D’Agostino et al., 2007, 2009). The high level of O_2_ increases O_2_-induced free radicals in the mitochondria, especially in complex I of the mitochondrial respiratory chain, which is particularly susceptible to reactive O_2_ species (Lenaz, 1998; Guo et al., 2013; Morales-Martínez et al., 2022). Hyperoxia can damage the activities of mitochondrial enzymes such as complex I and II and lead to mitochondrial dysfunction (Ramani et al., 2019; Machado et al., 2022). In addition, hyperoxia suppresses glucose transport, thereby causing local hypoglycemia and hypoglycemic failure (Wilson and Matschinsky, 2019). As a result of local hypoglycemia, K^+^, and extracellular excitatory amino acid neurotransmitters accumulate in the intercellular space (Palmer et al., 1994). Several studies have found that hyperbaric hyperoxia reduces the production of inhibitory neurotransmitters such as glycine and γ-aminobutyric acid (Hori, 1982; Gasier et al., 2017; Demchenko et al., 2023). We observed that the electrical excitability of putative type I interneurons in the CA3 decreased in hyperoxia, while the electrical excitability of putative type II interneurons increased in the CA3 region. The putative CA1 interneurons did not show a significant change in their firing properties. Neurons in the hilus are very sensitive to high O_2_ concentrations (Porzionato et al., 2015). In our previous study, where we investigated the effect of mild hypoxia, the inhibitory cells of the hilus reacted particularly sensitively to O_2_ deprivation, which was shown by a decrease in their firing frequency (Hencz et al., 2023). In this work, we did not report the firing frequency of the neurons of the hilus, because we had to discard most of the data due to the large spread in the ISI values. We hypothesize that lowered activation of inhibitory interneurons may lead to decreased regulation of principal cells in hyperoxia. Based on our results, the pyramidal cells and interneurons react differently to hyperoxic stimuli, and the functional heterogeneity of interneuron subtypes also reflects the effect of supplemental O_2_.

## Conclusion

In summary, the results of the present study reinforce previous observations that O_2_ can dose-dependently damage neurons in different regions of the hippocampus. In addition to the increased sensitivity of CA1 and hilus, we showed that CA3 neurons can also be damaged in a similar way as a result of short-term hyperoxia. The increased O_2_ level can also modify brain activity and stimulate a shift toward slow waves. The hyperoxic condition increases the excitability of the pyramidal cells, while probably suppressing the activation of a part of the putative inhibitory cells. The significance of the study is that it draws attention to the damaging effect of short-term mild hyperoxia. Therefore, the use of elevated O_2_ concentration inhalation in hospitals (i.e., COVID treatment and surgery) and in various non-medical scenarios (i.e., airplane emergency O_2_ mask, fire-fighters, high altitude trekkers, decompression chambers, military SCUBA divers, and “oxygen bars”) must be used with extreme caution.

## Data availability statement

The raw data supporting the conclusions of this article will be made available by the authors, without undue reservation.

## Ethics statement

The animal study was approved by the National Ethical Council for Animal Research (Permit number: BA/73/0052-5/2022, Hungary). The study was conducted in accordance with the local legislation and institutional requirements.

## Author contributions

AH: Formal analysis, Investigation, Visualization, Writing – original draft. AM: Data curation, Formal analysis, Methodology, Software, Writing – original draft. CT: Investigation, Writing – original draft. KK: Formal analysis, Investigation, Methodology, Writing – original draft. GS: Methodology, Writing – review & editing. JP: Formal analysis, Investigation, Methodology, Supervision, Writing – review & editing. AS: Conceptualization, Funding acquisition, Resources, Supervision, Writing – review & editing.
